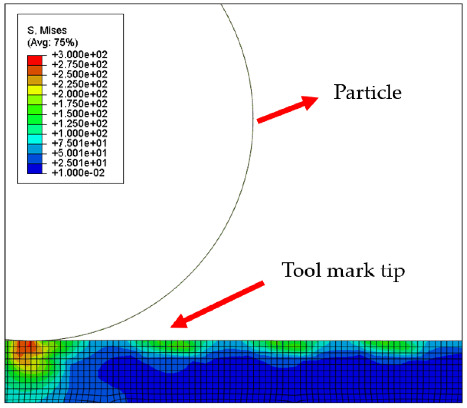# Modeling and Simulation of the Surface Generation Mechanism of a Novel Low-Pressure Lapping Technology

**DOI:** 10.3390/mi12121510

**Published:** 2021-12-04

**Authors:** Ninghui Yu, Lihua Li, Chea-su Kee

**Affiliations:** 1 Shenzhen Research Institute, The Hong Kong Polytechnic University, Shenzhen 518057, China; ninghui-a.yu@polyu.edu.hk; 2 Sino-German College of Intelligent Manufacturing, Shenzhen Technology University, Shenzhen 518118, China; 3 School of Optometry, The Hong Kong Polytechnic University, Hong Kong, China; c.kee@polyu.edu.hk

**Keywords:** ultraprecision lapping, Al6061, FEM

## Abstract

Aluminum alloy (Al6061) is a common material used in the ultraprecision area. It can be machined with a good surface finish by single-point diamond turning (SPDT). Due to the material being relatively soft, it is difficult to apply post-processing techniques such as ultraprecision lapping and ultraprecision polishing, as they may scratch the diamond-turned surface. As a result, a novel low-pressure lapping method was developed by our team to reduce the surface roughness. In this study, a finite element model was developed to simulate the mechanism of this novel lapping technology. The simulation results were compared with the experimental results so as to gain a better understanding of the lapping mechanism.

## 1. Introduction

**Figure 1:**
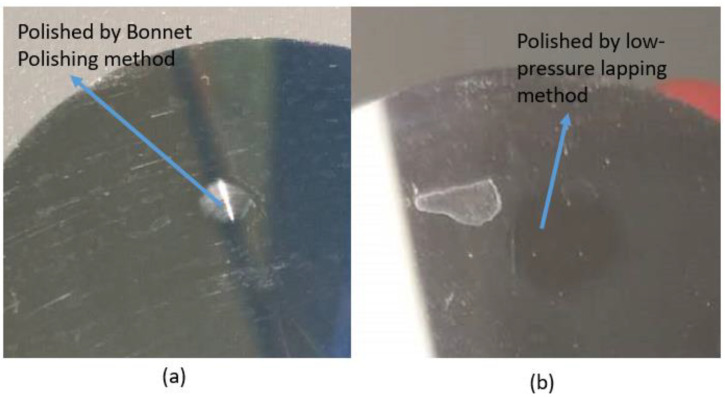
Result of Al6061 mirror surface polished by (**a**) Bonnet polishing method and (**b**) our low-pressure lapping method.

**Figure 2:**
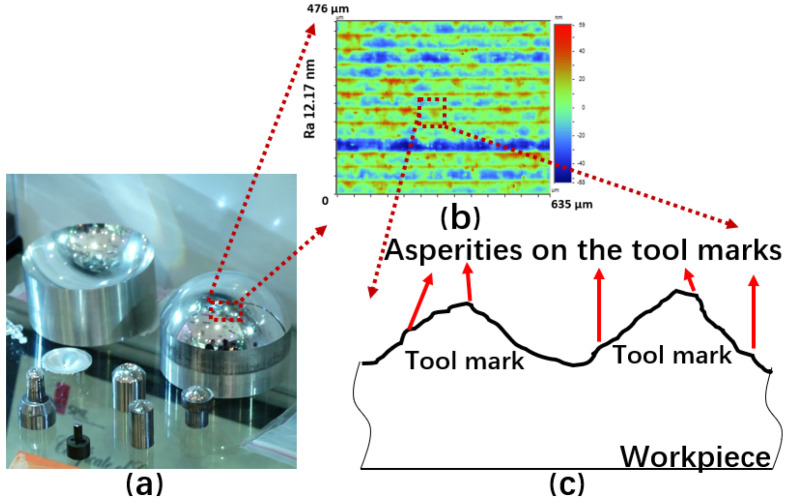
The aluminum alloy workpiece (**a**) looks shiny after SPDT but it actually has nanometer-level tool marks (**b**) on the surface. Asperities also exist on the tool mark (**c**).

Precision lapping and polishing are difficult for the polishing of softer metals such as aluminum alloys. As shown in Figure 1, many scratches occur on the aluminum alloy mirror surface after ultraprecision bonnet polishing. For aluminum and its alloys, single-point diamond turning (SPDT) is commonly used because of its good machinability. It is important to develop a method to remove the tool marks that are inevitably generated during the SPDT process (Figure 2). Hence, we developed a precision low-pressure lapping method for softer metals [1]. This method uses a brush to drive abrasive particles to roll and slide on the workpiece and has a very a small material removal rate as compared with other conventional precision lapping and polishing methods. The traditional lapping model based on the Preston formula is not suitable for this method. In the general lapping and polishing process, the common mechanisms are microcutting, delamination, slurry erosion, chemical mechanical polishing, etc. [2]. In our study, the brush touches the lapped surface very gently during the lapping process and drives the abrasive particles to move on the surface. The likely mechanism is that the abrasive particles receive large momentum during the lapping process. As a result, the abrasive particles impinge the asperities of the workpiece surface during the sliding and rolling process, causing plastic deformation of the asperities which are worn away, thereby reducing the surface roughness.

Since the asperities of the surface are removed by the impingement of the abrasive particles, the finite element method (FEM) can be used to model and simulate the surface generation in this low-pressure lapping. The simulation results provide a better understanding of the surface generation mechanism and can be used to design the lapping parameters of the actual experiment.

The finite element method (FEM) is an important analytical method for analyzing the deformation process of materials. Komvopoulus et al. used the finite element software Abaqus to simulate an area changing from elastic to plastic deformation under load [3]. This research shows that the material change process depends on strain hardening characteristics and accumulated plastic deformation at a specific load but has little to do with the elastic modulus. Kogut and Etsion studied the deformation process of the contact between a sphere and a rigid plane by the finite element simulation software ANSYS in 2002 [4]. The von Mises yield criterion was introduced to analyze the deformation process of the material, from elastic to plastic. The results showed that the elastic model cannot fully explain the elastic to plastic deformation process.

The basic contact model proposed by Greenwood and Williamson in 1966 is the basis of many subsequent microcontact models [5]. In this study, it is assumed that the contact surface is rough, the asperity heights on the surface follow a Gaussian distribution with the same curvature, and the asperities do not affect each other when they are under pressure. Contact in this model makes use of the Hertzian approach to calculate the pressure distribution during the contact process. The significance of this model is that it provides a preliminary contact model and makes use of a plasticity index that determines when the material transforms from elastic deformation to plastic deformation under a specific load.

Another classic model was established by Chang et al. based on the Chang, Etsion, and Bogy (CEB) elastic–plastic model by Tabor [6,7] and was the first attempt to determine the boundary state of a material from elastic to plastic. This model and the Greenwood and Williamson model provide good simulation for the contact process in a real contact area at very high or very low plasticity indices [5].

Using the Greenwood and Williamson model, Jackson and Green calculated the yield point of the material on the basis of the asperity contact and the von Mises criterion [8]. Kadin et al. found that the yield point of the material was greatly affected by Poisson’s ratio and strain hardening [9]. Peng et al. simulated the elastic–plastic contact process of rough surfaces by the finite element method [10]. The simulation model involves asperity contact with a rigid body. In 2017, Almuramady and Borodich conducted a theoretical and experimental comparison of the plastic behavior of an actual rough surface [11]. Although researchers have been continually studying the elastic–plastic contact of rough surfaces by finite element methods, they are rarely used to simulate the actual lapping process.

Since the core of the low-pressure lapping method involves abrasive particles continuously impinging on the tool marks, which are deformed and worn away gradually. In this study, the surface generation mechanism was simulated using current elastic–plastic contact models.

## 2. Modeling Processes

### 2.1. Parameter Setting

**Figure 3:**
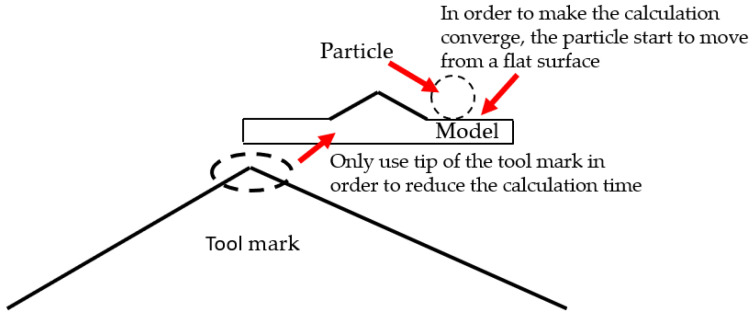
Schematic of the finite element model.

The simulation software Abaqus was used in this study. In the simulation, two different heights of tool marks on the Al6061 surfaces were designed to be 1 μm and 20 nm. In order to reduce the calculation time, only the tip of the tool mark was used, which means that the intercepted tool mark heights were 1 nm and 0.02 nm, respectively, in this simulation. Due to the surface with a tool mark height of 20 nm being almost close to the plane, in this simulation, it could be assumed that each tool mark was independent, and the movement of the abrasive particles on the surface was not affected by the adjacent tool mark. In this simulation, the tool mark was a long-ridged shape form; thus, when the abrasive grains collided from the side, it could be simplified into a 2D tool mark model. According to the experiment, the diameter of the abrasive particles in this simulation was 20 nm. In order to make the operation converge, a plane surface was added to the starting side, as shown in Figure 3.

Nanometer-level thermal expansion was identified to be an issue for some technologies such as thermal-assisted machining [12], while others take advantage of it for novel tool-displacement technologies [13]. Since the lapping speed in this research was low and the fiber gap on the lapping head helped to dissipate heat to some degree, this simulation only had a mechanical relationship and no variables such as temperature and magnetism were involved. Accordingly, the only material parameters needed to be set were mass density, elastic behavior, and plastic behavior. In the Abaqus software, there is no unit system, and it is necessary to set the unit according to the content of the simulation. To ensure the correct operation result, the unit setting needs to ensure consistency; otherwise, the results have no actual physical meaning. In Abaqus, units can be divided into two types: fundamental units and derived units. A derived unit is a combination of fundamental units. Since this simulation was in the micrometer range, the common meter-scale units were converted into micrometer units (Table 1) and were used in the simulation. The workpiece material in this simulation was aluminum alloy (Al6061) with a density of 2.7 × 10^−15^ kg/μm^3^, Young’s modulus of 6.89 × 10^4^ MPa, and yield stress of 276 MPa. In the assembly process, the initial position of the abrasive particle was on the right side of the tool mark, and the moving direction was from right to the left.

In the simulation, Al6061 alloy was considered as an elastic–plastic material according to the Johnson Cook plasticity model [14]. The model can be expressed as

(1) $$\bar{\sigma }=[A\;+\;B({\bar{\varepsilon }}^{pl}{)}^{n}][1+C\ln(\frac{{\dot{\bar{\varepsilon }}}^{pl}}{{\dot{\varepsilon }}_{0}})](1-{\hat{\theta }}^{m}),$$

where $\bar{\sigma }$ is the yield, ${\bar{\varepsilon }}^{pl}$ and ${\dot{\bar{\varepsilon }}}^{pl}$ are the equivalent plastic strain and plastic strain rate, and *A*, *B*, *C*, *n*, *m*, and ${\dot{\varepsilon }}_{0}$ are material parameters. When the material temperature is lower than the transition temperature, the coefficients are as shown in Table 2.

**Figure 4:**
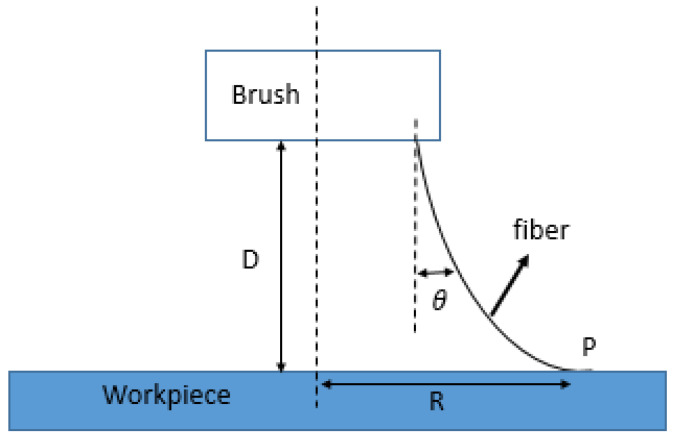
Schematic of the lapping process. One fiber on the brush is swept from the workpiece surface.

When the abrasive particles slide and roll on the surface of the workpiece, the polishing time is t, the rotational speed is S, the wool is bent after contact with the workpiece, and the angle of bending is θ. Since the diameter d of the thickest part of the wool is about 100 μm, one fiber sweeps N times on point P, which can be expressed as follows (Figure 4):

(2) $$N=\frac{2\pi (R-\tan\theta \cdot D)}{d}\cdot tS.$$

The speed at which the abrasive particle passes through the *P* point is

(3) v=2πκ·S·R,

where κ is a constant that represents how much of the fiber speed is transmitted to the abrasive particles.

The wear model for the nodes in this simulation was built based on the Archard model.

(4) $$q=\frac{kPA\eta }{H},$$

where q is the wear rate, k is a dimensionless constant obtained from experimental results and depends on several parameters such as surface quality and surface hardness process, P is the normal pressure, η is the interface slip rate, and H is the material hardness. In this simulation, the surface was expressed by the change of nodes on the surface. Each node transmitted force to the next node according to the software settings. The wear rate can be expressed as

(5) $$q(t)=\frac{k}{H}\int P(x,t)\eta (x,t)dA,$$

where x is the node position, and t is the time. Then, as a function of the Eulerian steady-state transport procedure [15], the model can change to a time-independent expression.

(6) $$q(t)=\frac{k}{H}\int P(u)\eta (u)T(u)du,$$

where u is the position along the edge of the grid, and T(u) is the width of the adjacent grid at position u. The wear rate can be expressed as a function of local material change.

(7) q(t)=∫w(u)T(u)du,

Since the software needs a discrete form expression, Equations (7) and (8) were combined and discretized as

(8) $$\sum_{i=1}^{N}w_{i}A_{i}=\frac{k}{H}\sum_{i}^{N}P_{i}\eta_{i}A_{i},$$

where w is the node wear velocity, and $A_{i}$ is the node contact area. The expression for w is

(9) $$w=\frac{k\sum_{i=1}^{N}p_{i}\eta_{i}A_{i}}{H\sum_{i=1}^{N}A_{i}}.$$

In this simulation, the momentum of the abrasive particles depended on the weight and speed of the abrasive particles. Since the individual abrasive particles were very small, the weight of the individual abrasive particles was about 5 × 10^−18^ kg according to the density of the abrasive material (2.65 g/cm^3^) and its size. From Equation (3), the different speeds of the abrasive particles when R is 5 mm were determined, as shown in Table 3.

### 2.2. Software Setting

**Figure 5:**
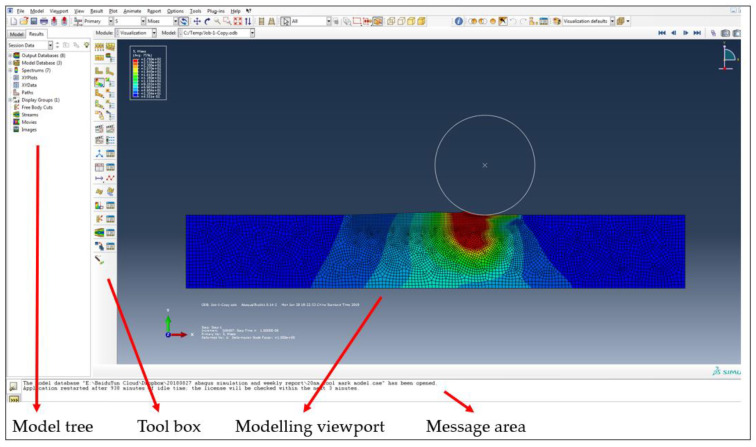
Two-dimensional model in Abaqus.

In the preprocessing stage, a two-dimensional model was first established in Abaqus, and the model was meshed (Figure 5). The boundary conditions and contact conditions of the contact model were then defined, and the abrasive particles were given an initial velocity. In the process of simulating the elastic–plastic contact between the abrasive particles and the surface of the workpiece, the contact process of the finite element simulation was a discontinuous constraint behavior, allowing the forces of the various nodes to be transmitted to other nodes. The premise of this constraint is that the two surfaces are to make contact. As a result, when analyzing the contact process, it is necessary to confirm that the two surfaces have been in contact and produced constraints.

Since the simulation was an impact process, the simulation made use of the Dynamic Explicit module from Abaqus. The software stores the simulation results as a binary file for the post-processing stage. In the post-processing stage, the simulation process can be visually displayed by the Abaqus software. At the same time, the basic variables can also be calibrated using the simulation results, such as displacement, stresses, and forces.

In the simulation, fatigue failure generally began with microcracks in the material. When the stress sufficiently accumulated in the structures, such as impurities, dislocations, etc., the material began to yield [16]. Generally, the fatigue crack is divided into three stages: initiation, propagation, and final fracture [17]. The early fatigue models were generally based on Hertzian contact to determine the maximum point of force to determine crack initiation. The recent rolling contact fatigue studies consider a rough surface as an important parameter [18,19].

Fatigue life is usually defined as a series of cycles or times that cause fatigue damage and cause a final failure [20]. In this simulation, the fatigue life was the stage at which the tool mark began to plastically deform after the abrasive grains repeatedly impacted on the tool mark.

## 3. Results and Discussion

**Figure 6:**
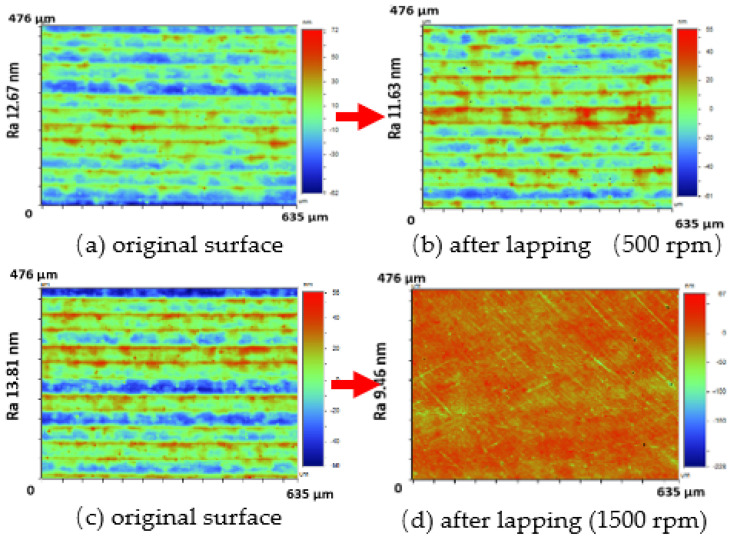
Two surfaces (**a**,**c**) with tool marks lapped under different rotational speed. The surface (**b**) had almost no change at a rotational speed of 500 rpm. The surface (**d**) started to show pits and scratches at a rotational speed of 1500 rpm [1].

Table 4 and Table 5 show the stress maps of the two different tool mark height models impacted by particles at different speeds. It can be seen from the two sets of simulation results that, when the abrasive grains were rolling and sliding at high speed on the surface of the workpiece, a greater speed led to a greater impact force on the surface. When an abrasive grain hit the tool mark fast enough, it bounced after the impact and was then pressed back by the fiber, repeatedly following the hit–bounce process. It can be seen from (d) and (e) in Table 4 and (d) and (e) in Table 5 that, as the momentum of the abrasive particles increased, the stress on the contact surface increased, and the distance between the two high stress areas became wider. This result indicates that a high rotational speed of the lapping pad does not mean a good lapping result for the actual lapping experiment. When the speed of the particle was high enough, the surface started to show pits and scratches because the particle impact on the surface was not even. Some particles may hit the surface to generate pits. Scratches may also be generated due to plowing by particles with large momentum. The experimental results also showed this phenomenon. We used a brush to lap the surface, and the diameter of the particles in the slurry was 15 nm [1]. As shown in Figure 6, after 5 min of lapping, the original surface (Figure 6a) had almost no change (Figure 6b) at a rotational speed of 500 rpm. However, there were pits and scratches generated on the lapped surface (Figure 6d) when the rotational speed was 1500 rpm (lapping time 20 min).

**Figure 7:**
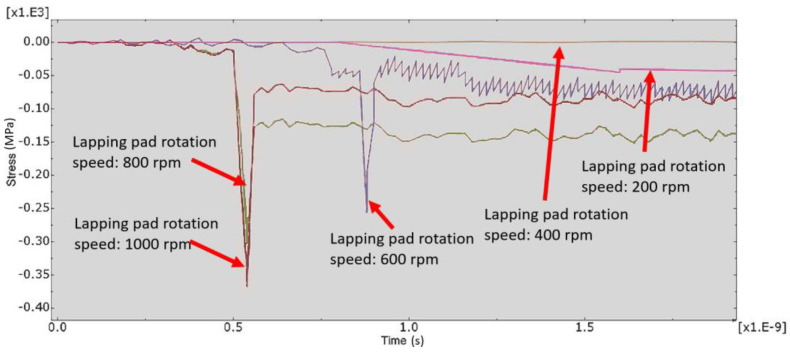
Stress–time curve of 1000 nm tool mark surface under different speeds.

**Figure 8:**
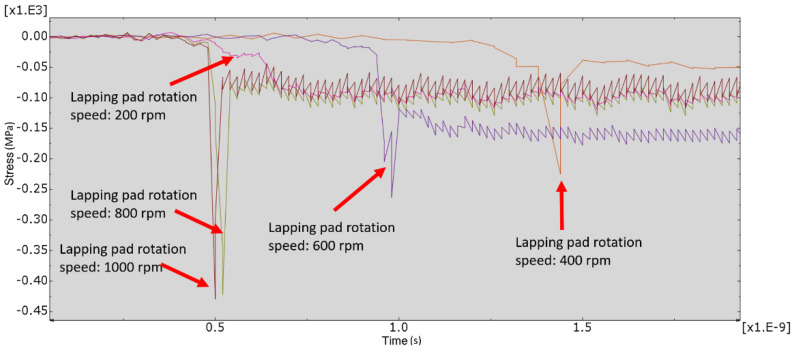
Stress–time curve of 20 nm tool mark under different speeds.

**Figure 9:**
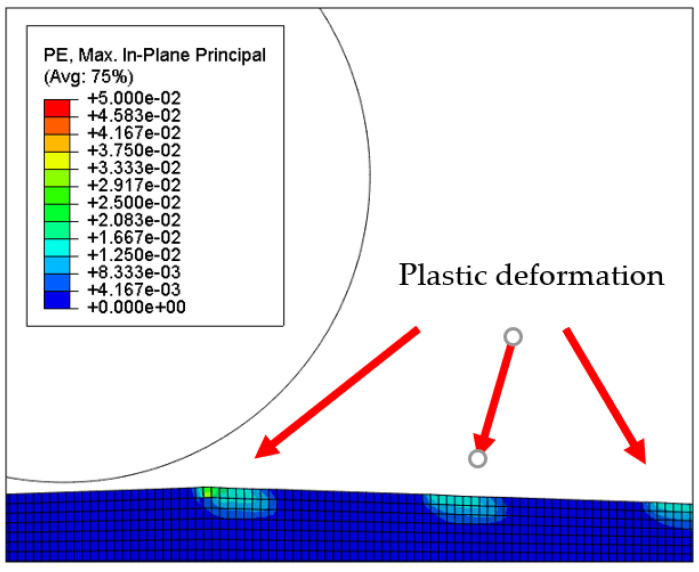
Plastic strain map of the 1000 nm high tool mark surface.

**Figure 10:**
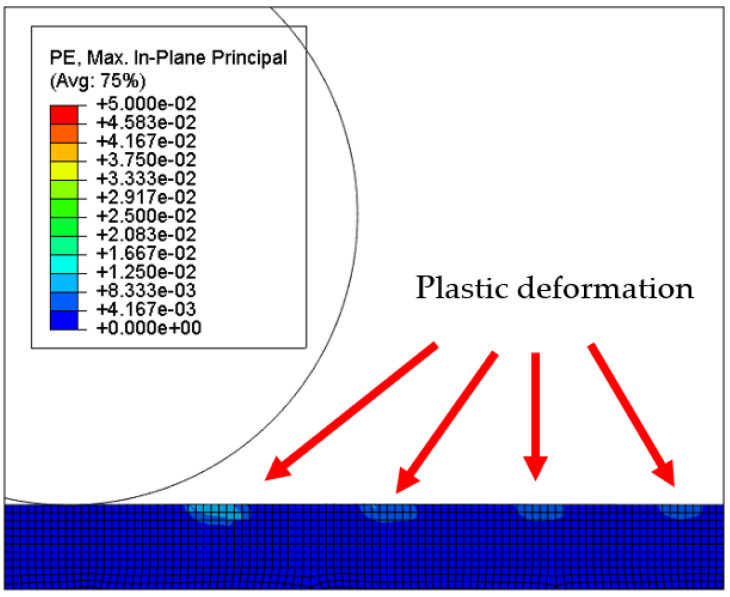
Plastic strain map of the 20 nm high tool mark surface.

Figure 7 and Figure 8 show the stress map of the tool marks under different impact speeds of the particles. Since the yield strength of the workpiece in this simulation was 276 MPa, it can be seen from Figure 7 and Figure 8 that, when the rotational speed was above 800 rpm, the stress of the abrasive grains in the simulation reached the material yield limit, and the contact area began to change from elastic deformation to plastic deformation. It can seen from Figure 9 and Figure 10 that, when the rotational speed reached 1000 rpm, plastic strain began to occur on the surface of the workpiece.

In the mechanical lapping process, the contact process between the particles and the workpiece determines the surface generation mechanism. Rubbing or plowing are widely accepted as the lapping material removal mechanism [21].

In this research, the diameter of the wool fiber was around 100 nm, and the diameter of the abrasive particle was 15 nm. Studies have shown that SiO_2_ particles are generally circular in the slurry [22]. As a result, the possible modes of interaction between a particle and the workpiece surface can be generalized into three categories:

Mode 1: The abrasive particles roll freely on the surface of the workpiece. The efficiency of material removal would be small, close to zero.

Mode 2: The abrasive particles plow a groove on the surface of the workpiece, and the material in the groove is extruded to the front and sides of the groove. In this case, the material removal rate is also close to zero, but the material stacked on the edge of the groove may be taken away by the subsequent abrasive particles.

Mode 3: The abrasive particle cuts a groove while a long strip of chip is removed from the groove. Ideally, the material is completely removed from the groove, and the material removal rate is close to 100%.

Mode 1 is usually called three-body abrasion, and the other two are generally known as two-body abrasion. Since the metal surface is soft, the abrasive particles move at a high speed under the action of the fiber and may be embedded in the workpiece surface, thereby changing from mode 1 to mode 2 or mode 3. However, since the material removal rate of this lapping method is very low, mode 1 plays the main role.

**Figure 11:**
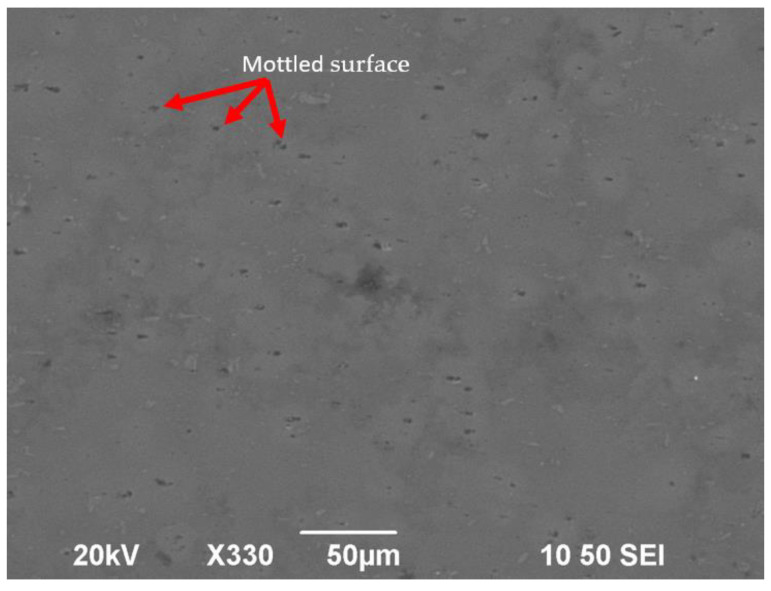
Mottled surface after lapping process (rotational speed 1000 rpm, time 15 min, slurry: SiO_2_).

**Figure 12:**
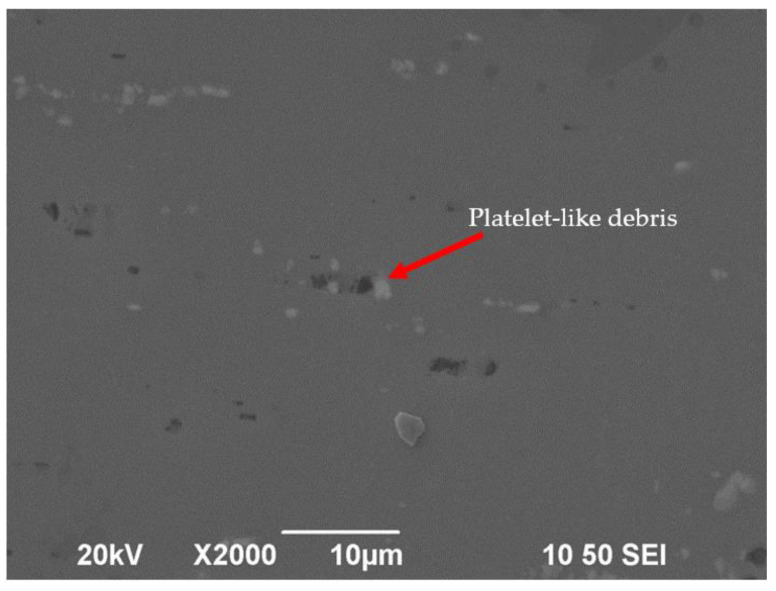
Platelet-like debris on the surface after lapping process (rotational speed 1000 rpm, time 15 min, slurry: SiO_2_).

It can be seen from the SEM image that some mottled surfaces were formed on the lapped surface (Figure 11), and some platelet-like materials were taken away during the lapping process (Figure 12). Some researchers believe that, when the abrasive particles are small, to a certain extent, such as less than 0.5 µm, the main mechanism in the lapping process is platelet delamination. However, it is still hard to explain why there was a wide range of scratch-free areas on the lapped surface.

**Figure 13:**
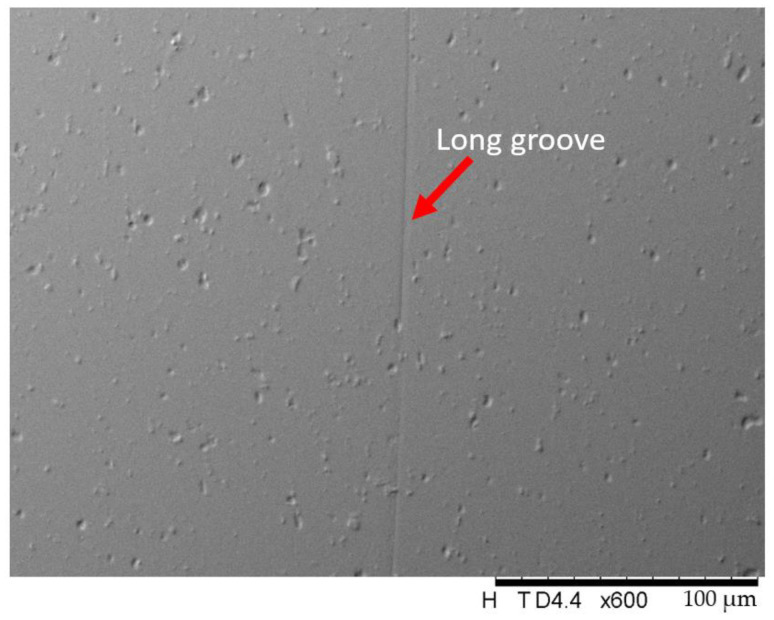
Long scratch on the lapped surface (rotational speed 1000 rpm, time 15 min, slurry particle: Al_2_O_3_).

For a single abrasive particle, it can microcut or plow the workpiece surface during the lapping process. However, as can be seen from the SEM image in Figure 13, a groove was rarely produced on the surface during the lapping process. In addition, the abrasive particles themselves were relatively round. As a result, the single-particle erosion mechanism only occupied a small proportion of the material removal in the low-pressure lapping process.

For the mechanism in which the abrasive particles are embedded on the fiber to form a cantilever system to establish a removal rate, the surface of the workpiece should have many shallow, long scratches. Although this method makes it is easier to lap a mirror surface, it can be seen from Figure 13 that only one shallow and long scratch existed. As a result, this mechanism is not suitable for this lapping method.

In the metal cutting process, there is a unique type of material removal known as extrusion cutting where there is a physical constraint ahead of the tool or particle [23,24] which may also have been involved in our lapping process. During the lapping process, many abrasive particles pass through the tool marks surface. When two abrasive particles pass through asperities, the front abrasive particles may provide a physical constraint, while the rear abrasive particles do the microcutting. In this scenario, the ductile mode cutting energy dominates [25], and plastic deformation occurs on the asperities, which is a mechanism worthy of further verification by experimentation.

Micromachining by abrasives contained in a carrier paste is a mechanism which has no microcutting behavior. This mechanism requires the lapping pad to be softer than the workpiece and the abrasive particles to be sufficiently small. At the same time, the abrasive particles should be easily embedded on the fiber. In this low-pressure lapping process, the fiber of the brush is far softer than the surface of the workpiece, while the abrasive grain is much smaller than the fiber; moreover, because the fiber has a good friction, the abrasive grain should be easily embedded on the fiber or driven by the fiber for high-speed motion. As a result, this mechanism is more suitable for the low-pressure lapping process. The high-speed movement of the abrasive particles means that there is sufficient momentum to laterally impact on the surface. When the momentum is accumulated to a certain level, the asperities on the surface of the workpiece are worn away, thereby reducing the surface roughness.

Through the above discussion, a possible mechanism for the low-pressure lapping method is that the fiber containing the carrier paste is worn away by the tiny asperities from the workpiece as the simulation illustrated in this paper. Although the simulation assumes that the speed of the abrasive particles is equal to the speed of the fiber on the lapping pad, the surface formation mechanism of the polishing method can be successfully verified from the simulation results. That is, the tool marks or asperities on the surface of the workpiece were deformed by the continuous impact of the abrasive grains and finally removed. The number of impacts was calculated according to Equation (3). When lapped for 10 min, the surface was impacted 3 × 10^6^ times. This is far more than the 10^5^ times of impact required for high cycle fatigue of typical materials. This cyclic stress is large enough to cause fatigue. As a result, it can be regarded as microscale high-cycle fatigue and can also be seen as impact fatigue for tool marks or asperities. These asperities and tool marks are worn away, and the worn surface continues to be impacted by the abrasive particles, which leads to the small-scale material removal rate.

**Figure 14:**
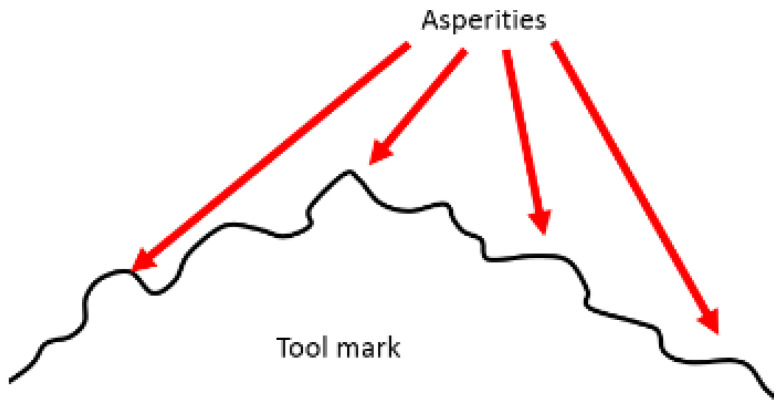
The asperities on the tool mark surface were removed first, followed by the tool mark itself.

According to our previous research results, there is almost no change in the lapped surface when the rotational speed is 500 rpm [1]. By finite element simulation, it was found that the rotational speed is the most important lapping parameter in this polishing method. When the rotational speed is lower than a certain level, it is difficult to achieve material removal. This also explains why the asperities on the tool mark surface were removed first, followed by the tool mark itself (Figure 14).

## 4. Conclusions

This research described the modeling and simulation of the surface generation mechanism of a novel low-pressure lapping method by the finite element method. The results indicate that the rotational speed plays a major role in the low-pressure lapping process. When the rotational speed was higher than a certain level, i.e., 1000 rpm in the experiment, the abrasive particles driven by the fiber obtained sufficient momentum to impinge on the surface of the workpiece. This finally wore the asperities and tool marks away. This model not only describes the surface generation mechanism of the low-pressure lapping method, but can also be used to predict the possible lapping parameters when different materials and forms of the workpiece are used. The recommended combination of lapping parameters determined from this simulation is to control the rotational speed at 800–1500 rpm and use particles smaller than the diameter of the fiber (i.e., 20 nm in this experiment).

## Figures and Tables

**Table 1:** Units used in the simulation.

Fundamental Units	
Length	mm
Force	μN
Time	s
Mass	kg
**Derived Units**	
Pressure	MPa
Velocity	μm/s
Density	kg/μm^3^
Young’s modulus	MPa
Yield stress	MPa

**Table 2:** Johnson Cook coefficients.

*A*	*B*	*C*	*n*	*m*	${\dot{\varepsilon }}_{0}$	$\theta_{melt}$	$\theta_{transition}$
289.6 MPa	203.4 MPa	0.011	0.35	1.34	1.0 s^−1^	925.37 K	294.26 K

**Table 3:** Speed of the particle in the simulation.

1	Rotational speed: 200 rpm	6.28 × 10^6^ μm/s
2	Rotational speed: 400 rpm	1.26 × 10^7^ μm/s
3	Rotational speed: 600 rpm	1.88 × 10^7^ μm/s
4	Rotational speed: 800 rpm	2.51 × 10^7^ μm/s
5	Rotational speed: 1000 rpm	3.14 ×10^7^ μm/s

**Table 4:** The 1000 nm high tool mark workpiece surface under particle impact at different speeds.

Tool Mark Height	Rotational Speed	Results
(a) 1000 nm	200 rpm	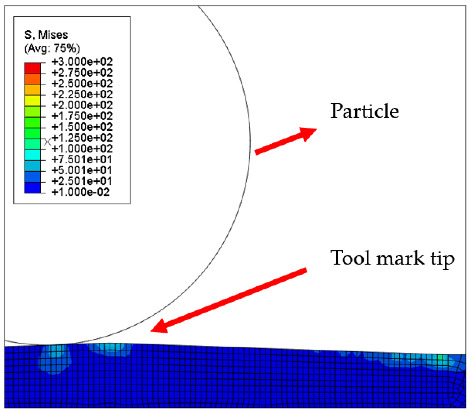
(b) 1000 nm	400 rpm	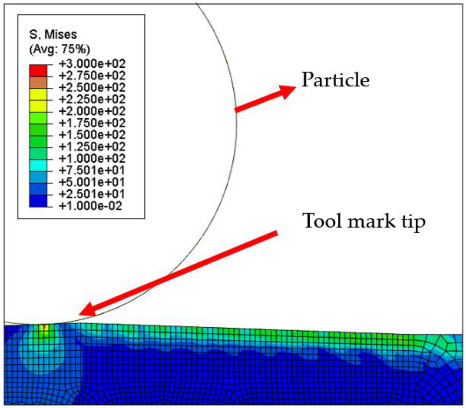
(c) 1000 nm	600 rpm	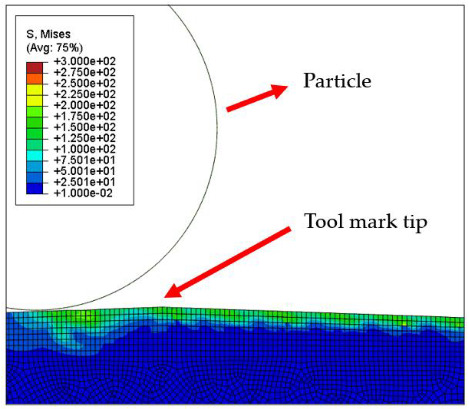
(d) 1000 nm	800 rpm	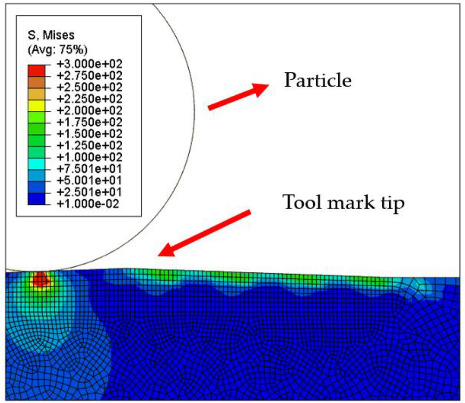
(e) 1000 nm	1000 rpm	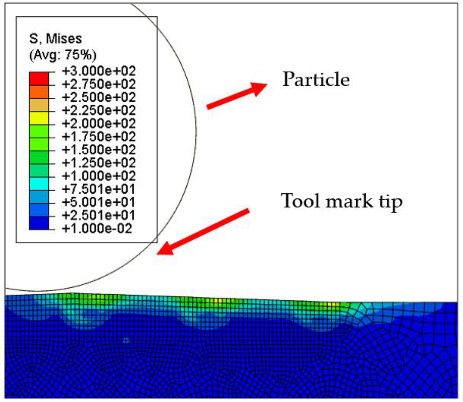

**Table 5:** The 20 nm high tool mark workpiece surface under particle impact at different speeds.

Tool Mark Height	Rotational Speed	Results
(a) 20 nm	200 rpm	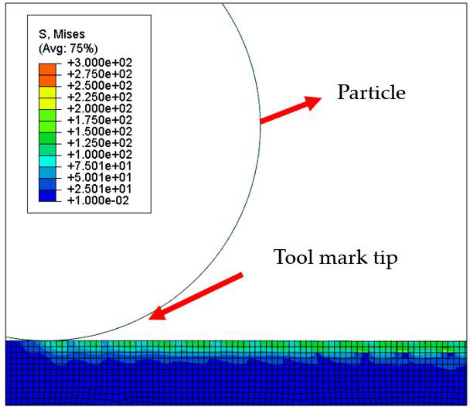
(b) 20 nm	400 rpm	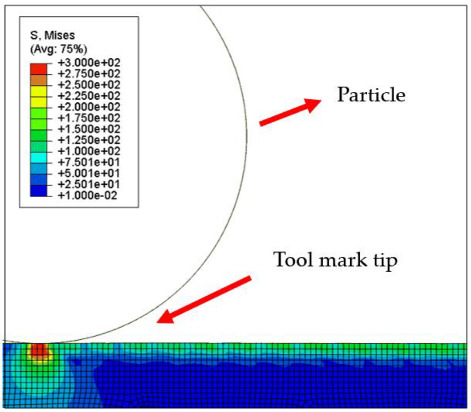
(c) 20 nm	600 rpm	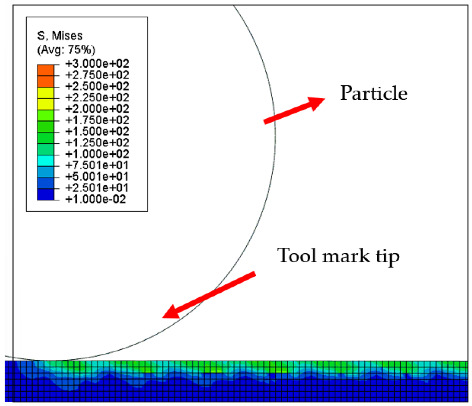
(d) 20 nm	800 rpm	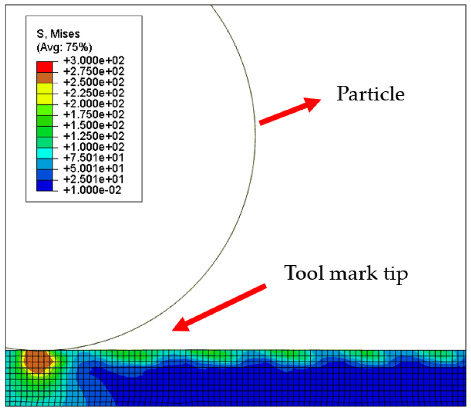
(e) 20 nm	1000 rpm